# Haematological nurses' experiences about palliative care trajectories of patients with life‐threatening haematological malignancies: A qualitative study

**DOI:** 10.1002/nop2.1558

**Published:** 2022-12-20

**Authors:** Siobhan McPherson, Ann‐Kristin Mitchell, Ida Sletten, Monica Evelyn Kvande, Simen Alexander Steindal

**Affiliations:** ^1^ Lovisenberg Diaconal University College Oslo Norway; ^2^ Department of Haematology Oslo University Hospital Oslo Norway; ^3^ Oslo Myeloma Center Department of Haematology Oslo University Hospital Oslo Norway; ^4^ Department of Anaesthesiology and Surgery University Hospital of North Norway Tromsø Norway; ^5^ Faculty of Health Studies VID Specialized University Oslo Norway

**Keywords:** haematological malignancies, haematology, nursing care, palliative care, qualitative research

## Abstract

**Aims:**

To explore haematological nurses' experiences about the palliative care trajectories of patients with life‐threatening haematological malignancies.

**Design:**

A qualitative study with a descriptive and explorative design.

**Methods:**

Data were collected through 12 individual semi‐structured interviews of nurses who work with patients with haematological malignancies from four hospitals in Norway. The data were analysed using systematic text condensation. The study was reported according to the Consolidated Criteria for Reporting Qualitative Research checklist.

**Results:**

Three categories emerged from the data analysis: focus on a cure delays integration of palliative care, dialogue with patients facilitates palliative care and the need for enhanced interdisciplinary understanding.

**Patient or public contribution:**

No patient or public contribution since nurses' experiences were explored.

## 1 INTRODUCTION

There have been important advances in the treatment of patients with haematological malignancies over the past few decades. However, many patients still die from their diseases or treatment complications (Wedding, 2021). Haematological malignancies are a group of blood cancers with heterogenous disease trajectories that can be broadly categorized into four subgroups: Hodgkin's lymphoma, non‐Hodgkin's lymphoma, multiple myeloma and leukaemia (Krok‐Schoen et al., 2018; Wedding, 2021). Haematological malignancies caused approximately 7% of all global cancer deaths in 2020 (Sung et al., 2021). This number is expected to keep increasing as the world's population continues to age as haematological malignancies have a higher prevalence and mortality rate in those over 65 years of age (Krok‐Schoen et al., 2018). The increasing prevalence of haematological malignancies indicates that a large proportion of these patients could have an extensive need for palliative care (Wedding, 2021). In a recent consensus‐based definition, palliative care has been defined as the active, holistic care of individuals with serious health‐related suffering because of severe illness, and it aims to improve the quality of life of patients, their families and their caregivers. Palliative care is applicable throughout the course of an illness in conjunction with treatment (Radbruch et al., 2020).

Studies have shown that combining palliative care and oncological treatment has several advantages, such as improved survival and symptom control, less anxiety and depression, reduced use of futile chemotherapy at the end of life, a better quality of life for the patient and improved family satisfaction (Dowling et al., 2020; Elliott et al., 2021; Kaasa et al., 2018). However, research shows that patients with haematological malignancies are less likely to be referred to palliative care. For those who are, this tends to occur later in the illness trajectory when compared to patients with other types of cancer (Hui et al., 2014; Vanbutsele et al., 2019; Wedding, 2021). Patients with haematological malignancies also have a higher risk of dying in the hospital due to complications from aggressive treatments. As a result, palliative care is often provided in the haematology oncology unit instead of a palliative care unit or hospice unit (Elliott et al., 2021; Hui et al., 2014; Manitta et al., 2010).

## 2 BACKGROUND

Due to the many advantages of early palliative care in oncological treatment, both patients and healthcare services would undoubtedly benefit from this integration (Kaasa et al., 2018). There are several barriers to integrating palliative care into the overall care plan of patients with haematological malignancies. These include the patient's uncertain prognosis and unpredictable illness trajectory, clinical optimism due to new therapies and clinical trials and lack of awareness of palliative care services (Manitta et al., 2010; Wedding, 2021). However, there is little research available on palliative care for patients with haematological malignancies, and even less on their palliative care trajectories (Moreno‐Alonso et al., 2018; Wedding, 2021).

Previous studies have investigated haematology nurses' experiences with and perspectives on end‐of‐life care (Grech et al., 2018; McCaughan et al., 2019; McGrath & Holewa, 2006, 2007a, 2007b). A study showed that close clinician–patient bonds, delayed end‐of‐life discussions and barriers to discharge contributed to patients receiving end‐of‐life care and dying in the hospital (McCaughan et al., 2019). Other studies have explored haematology nurses' experiences to develop a model for end‐of‐life care for patients with haematological malignancies. This model describes how openness towards addressing death and an organization open to discussing palliative care services can facilitate the integration of palliative care into haematology (McGrath & Holewa, 2006, 2007a, 2007b). Studies also suggest that the lack of integration of palliative care in the medical treatment of patients with haematological malignancies, prevents a dignified end‐of‐life and leads to nurses reporting high levels of emotional distress and powerlessness (Grech et al., 2018; McGrath & Holewa, 2006).

To the best of our knowledge, no studies have investigated nurses' experiences with the palliative care trajectories of patients with haematological malignancies from the early stages of the illness. Consequently, the aim of this study was to explore haematological nurses' experiences about the palliative care trajectories of patients with life‐threatening haematological malignancies.

## 3 METHODS

### 3.1 Design

This study employed a descriptive and exploratory qualitative design, which allows for flexibility when investigating and developing new knowledge on clinical nursing research topics with limited coverage (Hunter et al., 2019). The data were collected through individual, semi‐structured interviews. This approach could give nurses time to reflect and express their experiences in their own words, and make it possible to study and understand the nurses' insight on palliative care trajectories about patients with life‐threatening haematological malignancies (Polit & Beck, 2021).

### 3.2 Participants and recruitment

Participants were recruited using purposeful sampling from the haematological wards of four hospitals at national, regional and local levels in the eastern and western regions of Norway. Purposeful sampling was chosen to gain varied and rich data about nurses' experiences (Polit & Beck, 2021). To be included in the study, the participants had to be Registered Nurses with a minimum of 2 years' work experience caring for patients with life‐threatening haematological malignancies.

A total of 12 nurses working in inpatient wards agreed to participate in the study: three from a local hospital, four from two regional hospitals and five from a hospital with both regional and national functions. The scope of patients treated at these inpatient wards ranged from those undergoing milder chemotherapy to those receiving stem cell transplantation. One nurse had postgraduate training in palliative care whereas eight nurses had some palliative care training during their postgraduate education (five in cancer care) or master's degree (three in advanced practice nursing). The sample is further described in Table 1.

**TABLE 1:** Characteristics of study participants

*N* = 12	
Age range (mean)	30–52^a^ (40.0)
Years of work experience as Registered Nurse (mean)	5.5–28.5^a^ (14.1)
Years of experience in haematology (mean)	5–28.5^a^ (13.6)
Bachelor of science in nursing	3
Postgraduate education Palliative care Cancer care	1 5
Master of science in advanced practice nursing	3

^a^ Range.

### 3.3 Data collection

The interviews took place between November 2021 and January 2022 and lasted between 28–56 min (average of 39 min). They were conducted at the participants' workplaces and were audiotaped.

A semi‐structured interview guide was used to facilitate reflection and dialogue with the participants. The guide covered multiple aspects of nurses' experiences with palliative care trajectories with a focus on competency, initiative, decision‐making, communication and cooperation. The participants were encouraged to talk about their encounters with both dignified and undignified illness trajectories and how working with this group of patients affected them (Appendix S1). A pilot interview was conducted with an experienced nurse from the first authors' workplace to ensure that the questions in the interview guide were adequately relevant and understandable. After the pilot interview, an additional question was included about how the nurses experienced working with patients with life‐threatening illnesses.

Each interview was conducted by two first authors (SM, AKM or IS) who had professional relationships through work with five of the participants. One author interviewed the participants while the other took notes and ensured that the topics in the interview guide were sufficiently covered. To build trust, the first authors introduced themselves and explained their role in the interview setting. They employed an open and curious attitude to encourage the informants to share their experiences (Brinkmann & Kvale, 2015). Probing questions such as “Could you tell us a little more?” “Can you give an example?” and “How did you experience that?” were asked to prompt the participants to elaborate upon their answers (Polit & Beck, 2021). During the interviews, the first authors restated or summarized their interpretations of the nurses' answers and then further questioned them to determine the validity of the interpretations. At the end of the interview, the nurses were given an opportunity to speak freely, reflect on the topics and state any last thoughts or additions they might have. Immediately after each interview, the first authors wrote down their primary impressions from the interview to grasp what was perceived as the most important themes.

### 3.4 Data analysis

The interviews were transcribed verbatim by the first authors and inductively analysed using systematic text condensation (STC) (Malterud, 2012). STC is a descriptive and explorative method consisting of an iterative four‐step process to decontextualize and analyse interview data to gain accurate, recontextualized essences of participants' experiences as they were narrated (Malterud, 2012).

The transcripts were first read several times to get an overview of the material and identify the preliminary themes guided by the aim of the study. Each first author identified six to eight preliminary themes. Through discussion, three preliminary themes from these were agreed upon: a curative focus downgrades palliative care, the need for increased patient involvement and early integration of palliative care. The transcripts were re‐read to identify meaning units that were then organized into three code groups. The first authors maintained flexibility in code labelling and continuously assessed if meaning units should be reallocated to other groups. The meaning units in each code group were further analysed and organized into two or three subgroups. Each subgroup was abstracted into a summarising text by condensing the associated meaning units. These summaries formed the basis of the development of an analytical text that resulted in three categories of data. The categories were reviewed for consistency and accuracy by comparing them with the original transcripts. The authors agreed on category headings that represented the core results of the study and served to highlight its key findings. An example of the process of analysis is shown in Table 2.

**TABLE 2:** Example of stepwise analysis from the unit of meaning to category using STC.

Meaning units	Subgroup	Category
We give input based on our observations, we also have a dialogue with the patients beyond the very limited timeframe the doctors are present for a visit, sometimes the patients express their fatigue, that they have had enough, and we have to communicate that to the doctors. (nurse 7) We spend a lot of time with the patient, discuss a lot with the patient and his or her relatives, and we can often get an impression of where they stand and what they want. (nurse 10) We see the patients many more hours than the doctors do. The patients may look ok during the visit of the doctor, but they are not. I believe the doctors are sometimes a bit surprised that we bring up palliative care, but it starts a process and after a while they agree with us. (nurse 9)	Nurse observations and competence are important for the patients' trajectories	The need for enhanced interdisciplinary understanding
To think holistic at a much earlier stage, bring in more resources and make a plan that reflects what the patient's goal is – not what our goal is. (nurse 4) In general, I experience that we talk about palliative care at the end when the patient is terminal or preterminal. (…) it could have been initiated a little earlier, because there are many aspects both the patient and relatives have to think about at the end, if things had been clarified earlier then it might have been better for everyone. (nurse 5) I firmly believe the nurse should ask the doctor about the patient's outlook (…) what is the plan, is further treatment advisable? (nurse 12)	Planning is necessary to ensure satisfactory patient trajectories	
More interdisciplinary venues where we could decide together, plan and establish a treatment plan (…) containing details about what the patient's wishes are going forward. (nurse 10) So, I think it would be wise that the nurses are included in the decision‐making process because a joint meeting would help the nurses understand the doctors' thought processes. (nurse 2) Patients with haematological malignancies are often very complicated cases. I think communication between us and the doctors is critical, and improvements here will benefit the patient as well. (nurse 5)	There is a need for interdisciplinary cooperation and platforms for information exchange	

The transcripts and analysis were not returned to the participants for comments or corrections.

### 3.5 Trustworthiness

The first authors are Registered Nurses with experience in palliative care in haematology. They agree with the observation that the strong focus on curative treatment in haematology leads to palliative care only being considered late in the disease trajectory. By identifying and discussing both individual and shared preconceptions on the matter, the first authors maintained a conscious, critical and reflective approach to data collection and interpretation (Polit & Beck, 2021).

Development of the interview guide, data collection process and data analysis was discussed with co‐authors with experience in palliative care and haematology to further improve credibility (Graneheim & Lundman, 2004). The first authors and co‐authors had regular discussions on the results of each step of the analysis process and shared their different perspectives on the interpretation and relevance of the data (Malterud, 2012). A shared consensus on the final analysis and categories was reached, and a logbook of important decisions was maintained for reflexivity (Polit & Beck, 2021).

The variation in the level of treatment administered to the patients in the respective hospital wards contributed to diverse experiences about palliative care trajectories and enhanced the credibility of the findings.

The transferability of the study was strengthened by describing the sample, data collection and analysis process. Descriptions of the findings were supplemented with key quotations to allow the reader sufficient insight to evaluate the relevance of the answers and insights to other healthcare contexts (Graneheim & Lundman, 2004; Polit & Beck, 2021). To ensure clarity in the reporting of the study, the Consolidated Criteria for Reporting Qualitative Research checklist was followed (Tong et al., 2007).

### 3.6 Ethical considerations

The study was approved by the Norwegian Centre for Research Data (NSD) (reference number: 151084) and the data protection officer at each hospital prior to data collection. To ensure that the nurses did not feel pressured to participate in the study, they were recruited by the ward nurse manager of the individual units who then conveyed the participants' contact details to the first authors. All participants provided signed consent for their involvement in the study after receiving written information about the project. This included an assurance that all their information would remain confidential, their participation was voluntary and they could withdraw their consent at any time without consequences (Beauchamp & Childress, 2019). Prior to beginning the interview, the first authors repeated the information about voluntary participation in the study. The data collected was stored securely in accordance with the guidelines by NSD.

## 4 FINDINGS

Three categories emerged from the data analysis: focus on a cure delays integration of palliative care, dialogue with patients facilitates palliative care and the need for enhanced interdisciplinary understanding. Categories and subgroups are described in Table 3.

**TABLE 3:** Description of categories and subgroups [Correction added on 18 March 2023 after first online publication: the first subgroup in this table has been updated.]

Category	Subgroup
Focus on a cure delays integration of palliative care	Focus on a cure prevents palliative care planning There is always new treatment to be tested, but when is enough enough?
Dialogue with patients facilitates palliative care	Lack of openness around death and palliative care Communication and information are crucial for patient participation
The need for enhanced interdisciplinary understanding	Nurse observations and competence are important for the patients' trajectories Planning is necessary to ensure satisfactory patient trajectories There is a need for interdisciplinary cooperation and platforms for information exchange

### 4.1 Focus on a cure delays integration of palliative care

Several of the nurses had worked with both oncological and haematological patients and reflected on the differences in the way palliative care trajectories were integrated for the two groups. In their experience, oncologists were more familiar with palliative care principles, and palliative care was introduced earlier in oncological patients' illness trajectories. Haematologists, on the other hand, often focused on a cure until the patients were dying. According to the nurses, haematologists were reluctant to introduce palliative care if there was even a slight chance that patients could recover from their cancer. They often wished to try out new medications and include patients in clinical trials, and patients and relatives often expected to be offered the newest treatments. Several nurses believed that the multitude of new treatment options made it ethically difficult to introduce palliative care. As one nurse elaborated:

It's typically brought up very close to the end when you are more or less dying, far too late, it's as if there's no knowledge that palliative care can last several years. Doctors seem unable to think of palliative care at the same time as giving treatment (…) and if we bring up the need for palliative care, we are often told that it isn't necessary, we aren't at that stage yet. (Nurse 4)

Two nurses emphasized that it is impossible to know in advance who will survive and that everybody should be given the opportunity to be cured. Some patients are still alive because they were offered that chance. However, many nurses felt that treatment continued for too long, which resulted in patients dying rapidly once their treatment ended or from treatment complications. The nurses felt that some doctors were better than others at drawing the line and deciding when enough is enough. They also admitted that nurses should take a more active part in raising doctors' awareness of the value of integrating palliative care, especially in the context of frail or older patients receiving high doses of chemotherapy and, in some cases, even a bone marrow transplant. The nurses questioned whether milder treatments might be a better alternative for some patients. Although such treatment would not cure the patients, it could give them a few more good months to live without the heavy burden of side effects from chemotherapy and frequent hospital stays. As one nurse put it:

I think that we sometimes treat them a little too long (…) the boundaries have already been pushed far in respect to age and treatment, and it's very difficult, I'm glad I'm not a doctor who needs to decide who should receive treatment and who shouldn't (….) and maybe we give a treatment with poor prospects, in the hope of curing them, and then we end up in situations where we almost feel that we ‘kill’ the patient with the treatment. (Nurse 8)

### 4.2 Dialogue with patients facilitates palliative care

Several nurses emphasized that dialogue characterized by honest information and attentive communication was important to enable patient participation. In their opinion, this could facilitate a palliative care trajectory in accordance with the patient's wishes. However, a few nurses admitted that the ward lacked a routine for talking to patients about their preferences from the time of diagnosis. Nurses stated that a lack of openness about the possibility of dying and alternative treatments to cure could result in little time for planning and coordinating care. As a result, the patient could sometimes be too sick to be discharged from the hospital in time. Some nurses felt that patients received sufficient information that enabled them to make informed decisions about their treatment. When it was apparent that the patient might not survive, the nurses and doctors were competent in preparing them and their families for the worst outcome. Other nurses felt that doctors typically “dragged their feet,” waited too long before discussing the patient's prognosis, and that they should give the patient and their family realistic information about the situation at a much earlier stage. These nurses were concerned that the patients were not sufficiently informed about the possible risks and complications of treatment, and therefore did not have enough knowledge to comprehend how sick they could become. As one nurse described:

After a while we understand that they haven't understood the consequences of what they have agreed to, and sadly we experience that when they get exhausted someone finally has the courage to ask them, and then the answer is that had I known what I know now I think I would have said no to treatment. (Nurse 4)

The nurses interviewed believed that they were highly competent at creating a dialogue about palliative care with their patients. At the same time, they described that doctors and nurses were hesitant to talk to patients about palliative care and the possibility of dying early in the course of their illness. As illustrated by one nurse:

In my opinion, we never think about palliative care from the beginning of treatment. We're very focused on the fact that, yes, it's a serious illness, but we're going to handle this. I think we rarely talk about death and say: you could die of this. OK, maybe we say it once, but then we don't talk about it anymore. (Nurse 11)

This could lead to delayed implementation of palliative measures and prevent patients from having the opportunity to express their end‐of‐life preferences. One nurse felt that patients, deep down, were aware of their risk of dying and that such conversations did not necessarily have to be unpleasant. Many nurses reflected on the challenge of determining the right time for such a conversation. On the one hand, they did not want to take away the patient's motivation for treatment and hope of survival, but on the other hand, they did not want to rob them of time at home with a good quality of life and a dignified death.

### 4.3 The need for enhanced interdisciplinary understanding

The nurses perceived that they were a step ahead of the doctors in identifying patients who might be eligible for palliative care. They said that they have a unique insight into the patients' physical and mental state and individual preferences because they care for them at all hours and in various situations. Many nurses pointed out that while having a specialization or degree was an advantage, experience was just as important to be able to identify those patients in need of palliative care. The nurses often attempted to share their observations with the doctors and described advocating the patient's case as an important part of their work. As one nurse described:

We try to put forward our points of view, and kind of, speak on behalf of the patient and his or her family. We try to suggest that it might be, purposeful is maybe not the right word, but important for the patient to have a good quality of life, or if we should continue with treatment that only torments them. (Nurse 6)

Some nurses expressed frustration when doctors did not listen to their assessments and functioned as gatekeepers for introducing palliative care. Many experienced nurses, on the other hand, felt that their voices were heard:

I think the dialogue is complementary: we are often the ones who bring it up, and then the doctors balance it against what they have experienced can work and what can't. (Nurse 3)

One nurse spoke of discussions between fellow nurses on how palliative care planning should be considered from the moment patients receive the diagnosis. The nurses believed that it was their duty to take an active part in the establishment and evaluation of treatment plans because patients with haematological malignancies often experience acute changes in health status. Several nurses described frustrating situations during weekends where a lack of planning often caused insecurity and decision aversion in doctors who did not have adequate knowledge of the patients' medical histories. The nurses believed that a long‐term treatment plan made by the haematologist in charge of the patient would allow for a shared understanding and prepare everyone involved. As one nurse said:

Maybe have a plan, that if this doesn't work, considering all the treatment, that we have a plan for when it isn't purposeful anymore, a plan for what we should do, together with the patient. That the patient also knows, is prepared maybe, for the possibility that if this doesn't work, then OK, we have reached a new phase. (Nurse 6)

The nurses maintained that it is the doctor's right to have the final say in the decision‐making process, as they have the ultimate responsibility for the patient. However, they also believed that there was much to gain from nurses contributing to the decision‐making process about palliative care, as their input could improve palliative care planning.

Nurses argued that there was a lack of arenas for information exchange between nurses and doctors. Segregated planning meetings were perceived as a barrier to cooperation. Furthermore, several nurses found it challenging to speak up during their daily meetings with the haematologists due to time constraints and doctors dominating these meetings. Nurses stressed how important it is to understand the reasons behind doctors' decisions about the implementation of palliative care, given that explaining medical and treatment decisions to patients was often the nurses' responsibility. To ensure satisfactory patient trajectories, the nurses expressed the need for weekly interdisciplinary meetings where they could discuss complicated patient cases. The nurses hoped that such meetings could contribute to a common understanding of the treatment objectives and expected outcomes and allow for ethical reflections on palliative care trajectories. One nurse shared positive experiences of interdisciplinary cooperation:

We discuss amongst ourselves since we experience things differently and have contrasting standpoints (…) the focus is on working as a team and not as separate professions, which benefits the entire ward. (Nurse 11)

## 5 DISCUSSION

This study gives insight into nurses' experiences about palliative care trajectories of patients with life‐threatening haematological malignancies. The findings suggest that the integration of palliative care is hindered by a curative focus because there may be a chance that patients can recover. The nurses experienced a lack of openness about death and believed that enhanced dialogue with patients and interdisciplinary cooperation between doctors and nurses could improve palliative care trajectories.

Our participants believed that patients should have the opportunity to benefit from medical progress and new treatments, as they could lead to an improved chance of survival or prolonged life. Nevertheless, in line with previous studies (Grech et al., 2018; McCaughan et al., 2019; McGrath & Holewa, 2006), nurses in our study experienced that this medical focus delayed the integration of palliative care into treatment pathways. In haematology, where the treatment goal is primarily to cure or prolong life, there might be insufficient knowledge of the benefits of early integration of palliative care (El‐Jawahri et al., 2020; Kaasa et al., 2018). However, patients with haematological malignancies already have extensive palliative care needs from the time of initial diagnosis, and throughout their illness trajectory, as they receive intensive treatments such as stem cell transplants and high‐dose chemotherapy regimens with a high risk of toxicity and mortality (El‐Jawahri et al., 2020).

Our participants experienced the doctors' decisions on whether to continue treatment as ethically difficult and that some treatments continued for too long or were futile. The impression that patients were treated more aggressively than the participants believed should be the case could be a source of moral distress for the participants. Sanderson et al. (2019) define moral distress as “ethical unease or disquiet resulting from a situation where a clinician believes they have contributed to avoidable patient or community harm through their involvement in an action, inaction or decision that conflicts with their own values.” A quantitative literature review found that nurses experienced moral distress more frequently when they acted in a way that they felt was not in the patients' best interest such as providing futile care (Oh & Gastmans, 2015). According to Benner et al. (2011), the decision to continue or terminate treatment needs to be based on both ethical and clinical reasoning, as it is unethical to either give futile care or not offer patients the best available treatment. Reports from the Lancet Oncology Commission and Lancet Commission on Value of Death indicate that patients and doctors tend to focus on treatment in uncertain situations due to their hope for prolonged survival (Kaasa et al., 2018; Sallnow et al., 2022). Furthermore, offering curative therapies seems to have become synonymous with caring for patients (Benner et al., 2011). Our participants felt that the treatment sometimes continued for too long and questioned its aggressive nature especially when administered to frail patients. The option of not treating certain patients should be actively considered (Benner et al., 2011) as new treatments often extend life only marginally, and end‐of‐life chemotherapy produces more harm than good in patients over 80 years of age (Krok‐Schoen et al., 2018; Sallnow et al., 2022).

Our participants had different experiences with how well patients were informed, and some believed that a lack of timely and honest information hindered open dialogue with patients. Honest discussions, even when the prognosis is poor, could enhance the relationship between patients and the medical team. However, avoiding prognostic discussions could lead to mistrust, anxiety, reduced quality of life and family distress (Kaasa et al., 2018). The nurses in our study believed that explaining different scenarios prepared patients for what was to come and gave them the oppourtunity to participate and make informed decisions about their treatment and care throughout the illness trajectory. This is concurrent with the results of a study indicating that early, frank discussions with the patients and their families about likely treatment outcomes could avoid unrealistic expectations (McCaughan et al., 2019). Prognostic outcomes when treating haematological malignancies are often hard to predict, and it can be challenging for patients to decide whether undergoing aggressive treatment is worth the suffering involved (Wedding, 2021). A lack of understanding of their prognosis could make patients overestimate the chance of a cure and is associated with an increased willingness to accept chemotherapy (El‐Jawahri et al., 2020). Even if patients have received adequate information about their disease and prognosis, they may not be able to understand the intention of the treatment (Sallnow et al., 2022) or remember the information later (Wedding, 2021). Kaasa et al. (2018) underlined the importance of assessing what patients already know and the level of detail that they want and using non‐technical language when explaining the prognosis, encouraging questions, verifying their understanding and tailoring communication to meet the patient's needs.

Our study suggests that the nurses regard themselves as competent in discussing palliative care with patients, however, in line with the study by McCaughan et al. (2019), they hesitated to discuss death and found it challenging to find the right time for these conversations. According to our participants, this could prevent patients from expressing their end‐of‐life wishes. Each patient has individual beliefs, values and needs that should be reflected in their care (Österlind & Henoch, 2021). Through open, two‐way dialogue, nurses can enhance patients' ability to express how they view and experience their situation and preferences and enable them to be co‐creators of their palliative care trajectory. This person‐centred care approach allows the nurses to help patients live as good a life as possible during their palliative care trajectory (Österlind & Henoch, 2021). Such discussions are essential and should be seen as a professional responsibility throughout the illness trajectory (Sallnow et al., 2022). Nurses sometimes experience human and nursing failure in care when patients do not have time to prepare for death. Therefore, it is important that nurses work towards bridging this gap by discussing human aspects of illness and hospitalization, such as the patient's concerns, fears and hopes (Benner et al., 2011).

To meet the complex needs of patients with life‐threatening diseases, palliative care is often provided by multidisciplinary teams with professionals from different disciplines (Leclerc et al., 2014; World Health Organization, 2016). In contrast, patients with haematological malignancies primarily receive palliative care from their haematology team (Wedding, 2021). Our participants believed that an enhanced interdisciplinary approach, where doctors and nurses discuss patients and share their assessments, can lead to a common understanding and more holistic palliative care trajectories. Interdisciplinary cooperation entails that professional groups share knowledge and plan patient care together (Klarare et al., 2013; Leclerc et al., 2014). However, our participants reported a lack of opportunities and arenas to meet and discuss with doctors to allow for this exchange. This caused frustration in the nursing team. Poor teamwork and team support, such as difficulties in maintaining open communication and a collaborative environment, may also lead to moral distress among healthcare professionals (Maffoni et al., 2019; McCarthy & Gastmans, 2015). The integrative review by Mafoni et al. (2019) suggests that collaborative and respectful relationships and opportunities to have confrontations and exchange of experiences may help healthcare professionals to cope with moral distress.

Our participants described that doctors and nurses had different understandings of the palliative needs of patients with haematological malignancies. Nurses spend considerable amounts of time with their patients and can therefore contribute with vital medical information and the patient's perspective. In line with previous studies (Grech et al., 2018; McCaughan et al., 2019), our participants described advocating the patient's needs and concerns as being an important nurse responsibility. This can be explained by the fact that most participants in our study were experienced nurses. Because of their knowledge of the patients' needs and concerns, our participants believed that they could influence and improve palliative care planning. Their engagement in establishing and updating treatment plans was important for establishing a common understanding in the medical team. In accordance with the study by Grech et al. (2018), our participants saw the need to establish a treatment plan from the time of initial diagnosis, which should be a standard approach for all oncology patients (Kaasa et al., 2018). A treatment plan containing prognostic information and guidelines for different trajectories could be beneficial to organizing better clinical care. Furthermore, such a plan could help patients and their families better understand and cope with their situation (Kaasa et al., 2018).

### 5.1 Limitations

The ward managers of the different wards had sole hands in recruiting the participants and may have done so in a manner to represent their own or the ward's views about palliative care trajectories. Additionally, they could have chosen participants who they knew would represent the ward in a favourable manner.

A sample of 12 nurses might be considered small. However, the participants had diverse experiences and provided rich data related to our study's aim. We perceived that during the interviews, the nurses shared their reflections and experiences openly and honestly. The sample size was therefore considered to have sufficient information power (Malterud et al., 2016). Participants were selected from both the eastern and western parts of Norway, and a majority of the nurses had extensive clinical experience; all except three had a postgraduate or master's degree that included training in palliative care. Nurses from other geographical parts of Norway, novice nurses and nurses without postgraduate training in palliative care could have had other experiences or opinions on the matter.

Nurses who were familiar with the first authors and the established social norms at their mutual workplace may have been hesitant to talk about sensitive topics or challenge institutional rules and conventions. They may also have made assumptions about the first authors' preconceptions, goals and knowledge on various topics, which might have influenced the answers that they provided (McEvoy, 2002). However, these interviews were conducted by the first authors who were least acquainted with the respective participants to encourage the participants to express their thoughts more freely. When reviewing the interviews of the nurses in question, they were found to share experiences that were both critical and supportive of current practices.

The interviews were conducted by two first authors, which may have affected the participants' ability to relax and feel comfortable providing candid, genuine answers since they were in the minority during the interview (Polit & Beck, 2021).

## 6 CONCLUSION

The nurses experienced that the integration of palliative care was hindered by a medical focus aiming to cure or prolong life, which could lead to patients being overtreated. The nurses described a lack of openness about the oppourtunity of palliative care and death. They believed that enhanced dialogue with patients would allow them to better understand their prognosis, include them in treatment decisions, and give them time to prepare for death. The nurses also believed that enhanced interdisciplinary cooperation could improve long‐term planning and, subsequently, patients' palliative care trajectories. However, they experienced a lack of arenas where they could share their assessments and discuss their patients with the doctors. Nurses' insights on patients' needs and concerns could contribute to more holistic palliative care trajectories and ensure a person‐centred approach to care. Future research should therefore focus on ways to improve collaboration between nurses and doctors working in haematology wards. Based on our findings, we would also recommend further research into the development of palliative care guidelines, which incorporates knowledge from both haematologists and haematological nurses. Involving patients and relatives in the development of these guidelines can further improve patients' illness trajectories.

## 7 RELEVANCE TO CLINICAL PRACTICE

To improve the palliative care trajectories of patients suffering from life‐threatening haematological malignancies, there is a need for increased openness and dialogue around death and palliative care from the time of initial diagnosis. Haematologists need to be made aware of the benefits of palliative care and how it can be applied in conjunction with standard treatment throughout the patient's illness trajectory. Furthermore, arenas that facilitate the exchange of observations and assessments between doctors and nurses are vital to improving palliative care planning. Establishing such arenas should therefore be a priority. Consequently, palliative care should be included in the official guidelines for patients with haematological malignancies, and internal procedures to secure holistic palliative care trajectories must be established in the wards.

## AUTHOR CONTRIBUTIONS

Design: SM, AKM and IS; data collection and drafting of the manuscript: SM, AKM and IS; data analysis: SM, AKM, IS, MEK and SAS and critical reviewing of the manuscript: MEK and SAS. All authors approved the final version of the manuscript.

## FUNDING INFORMATION

SM, AKM and IS wrote this article as part of their master's degree which was funded by the Department of Haematology, Oslo University Hospital, Oslo, Norway.

## CONFLICT OF INTEREST

The authors declare no conflict of interest.

## Supporting information


**Appendix S1.** Supporting Information.

## Data Availability

The data that support the findings of this study are available on request from the corresponding author. The data are not publicly available due to privacy or ethical restrictions.
