# Supplementary material for: Haematological nurses' experiences about palliative care trajectories of patients with life‐threatening haematological malignancies: A qualitative study
Source: Nurs Open. 2022 Dec 20;10(5):3094–103. doi: 10.1002/nop2.1558 (PMC10077405; doi:10.1002/nop2.1558)
Supplement: Supplementary file 1 — Appendix S1. Supporting Information. [file NOP2-10-3094-s001.docx]

**APPENDIX**

**Interview guide**

Please note that this guide represents only the main themes discussed with the participants and does not include probing questions such as “Could you tell us a little more?” and “Can you give a typical example?”.

**Introduction**

Thank you for agreeing to participate in this study.We are interviewing you to better understand haematological nurses’ experiences regarding palliative care trajectories of patients with life-threatening haematological malignancies. When we refer to experience regarding palliative care trajectories, we mean experiences and viewpoints around the process of initiation and implementation of palliative care and palliative treatment for this group of patients. There are therefore no right or wrong answers to the questions we ask you. It is important for us that different points of view are represented. Depending on how much information you would like to share, we expect the interview to last approximately 1 hour. With your permission the interview will be recorded on audiotape, and we will be taking notes during the interview. Remember that in order to maintain confidentiality during the interview, it is important that the information you provide cannot be recognised. If you talk about people who are not present in the room, do not use their names.

**Establishing Rapport**

Before we begin, it would be nice if you could tell us a little about your background as a nurse and your work experience.

What does palliative care signify for you?

When in the patient’s illness trajectory is palliative care and palliative treatment typically initiated and implemented in your workplace?

- What promotes implementation?

- What prevents implementation?

How does the decision-making process typically look where you work, in terms of limiting treatment of patients with life-threatening haematological malignancies?

- In your opinion, how can nurses contribute to the decision-making process?

What is the nature of the cooperation between doctors and nurses where you work, when it comes to palliative care and palliative treatment to patients with life-threatening haematological malignancies?

- What works well?

- Is there anything that should be changed?

What skills do you think nurses need in order to follow up patients with haematological malignancies in a palliative care trajectory?

- Who do you turn to if you need advice and guidance?

Can you tell us about a typical situation where a patient who died of a haematological malignancy had a palliative care trajectory that was dignified?

- What made this trajectory dignified?

Can you tell us about a typical situation where a patient who died of a haematological malignancy had a palliative care trajectory that was undignified?

- What made this trajectory undignified?

Do you get affected by working with patients with uncertain prognosis and potentially short life expectancy?

- If so, in what ways?

What are your thoughts about the current practices at your workplace regarding palliative care trajectories of patients with haematological malignancies?

- What works?
- Is there anything that should be changed?

**Conclusion**

Is there anything you wish to add? Is there anything we haven’t asked you about that you think is important to include?

Thank you for your participation!
